# Eosinophilic gastroenteritis as a cause of non-*Helicobacter pylori*, non-gastrotoxic drug ulcers in children

**DOI:** 10.1186/s12876-020-01416-7

**Published:** 2020-08-20

**Authors:** Jung Yeon Joo, Jin Min Cho, In Hyuk Yoo, Hye Ran Yang

**Affiliations:** 1 Department of Pediatrics, Seoul National University Bundang Hospital, Seoul National University, 82, Gumi-ro 173 Beon-gil, Bundang-gu, Seongnam-si, Gyeonggi-do 13620 South Korea; 2 Seoul National University College of Medicine, Seoul, South Korea

**Keywords:** Peptic ulcer, Eosinophilic gastroenteritis, Eosinophilia, *Helicobacter pylori*, Non-steroidal anti-inflammatory agents, Child

## Abstract

**Background:**

While *Helicobacter pylori* (*H. pylori*) ulcers has declined recently, *H. pylori*-negative and/or gastrotoxic drug-negative peptic ulcers (HNGN-PU) has increased. This study aimed to analyze the etiology of peptic ulcers in children and the differences in clinical, laboratory, endoscopic, and histopathologic findings of peptic ulcers according to etiology, including eosinophilic gastroenteritis (EoGE).

**Methods:**

In total, 255 children (157 boys and 98 girls) with peptic ulcers were recruited. The subjects were categorized into 5 groups according to the etiology of the ulcer: 1) *H. pylori* infection (*n* = 51); 2) gastrotoxic drugs (*n* = 18); 3) idiopathic (*n* = 144); 4) systemic disease (*n* = 23); 5) EoGE (*n* = 19). Clinical data were reviewed and analyzed retrospectively.

**Results:**

Age at diagnosis, ulcer recurrence, atopic dermatitis history, white blood cell count, blood eosinophil count, platelet count, serum albumin level, iron level, erythrocyte sedimentation rate, and C-reactive protein level differed significantly among the 5 groups (all *p* < 0.05). Regarding endoscopic findings, multiple ulcers and gastric mucosal nodularity differed among the 5 groups (all *p* < 0.05). When comparing the EoGE ulcer group with the others, EoGE group revealed older ages (*p* = 0.022), higher rates of ulcer recurrence (*p* = 0.018), atopic dermatitis history (*p* = 0.001), and both blood and tissue eosinophilia (both *p* = 0.001).

**Conclusions:**

EoGE ulcers constituted 10.2% of HNGN-PU in pediatric patients. In children with HNGN-PU, peripheral eosinophilia, ulcer recurrence, and atopic dermatitis history might imply EoGE, necessitating thorough investigation of tissue eosinophils during endoscopic biopsy.

**Trial registration:**

A total of 255 children was retrospectively registered between between July 2003 and April 2017.

## Background

Peptic ulcer disease is mainly associated with *Helicobacter pylori* (*H. pylori*) infection [1–4], and the use of gastrotoxic drugs such as non-steroidal anti-inflammatory drugs (NSAIDs) [5]. However, in the last decade, the main etiology of peptic ulcer disease has changed significantly in both children and adults from both eastern and western countries [6–8]. The prevalence of both *H. pylori* ulcer and gastrotoxic drug peptic ulcer has declined due to improved eradication therapy for *H. pylori* infection and reduced use of NSAIDs, respectively [9–11], while there have been several reports on the recurrence of peptic ulcers after the eradication of *H. pylori* in non-users of NSAIDs [6, 12, 13]. Thus, *H. pylori*-negative and/or gastrotoxic drug-negative peptic ulcer (HNGN-PU) has emerged as a “new” disease entity [14, 15].

Most cases of *H. pylori*-negative peptic ulcers are caused by NSAIDs in adults [5], but not in children. Although the prevalence of HNGN-PU is reported to be higher in children than in adults [6, 16], the exact etiology of HNGN-PU in children is unclear and has not been investigated yet.

Eosinophilic gastroenteritis (EoGE) is an inflammatory disorder characterized by eosinophilic infiltration of the stomach and/or duodenum. In some cases, inflammation of the esophagus, distal intestine, and colon may also be present. EoGE occurs without any other known cause of tissue eosinophilia. Vomiting, abdominal pain, and growth retardation are the most common symptoms [17], and approximately 40% of patients with EoGE have a history of allergic disease including asthma, eczema, or rhinitis [17–19]. Previous studies in adult patients have reported EoGE to be a cause of intractable peptic ulcers in some patients. However, there have been no studies investigating EoGE as the etiology of peptic ulcers in children yet, even though EoGE can be a potential etiology of HNGN-PU in children.

The present study was aimed at analyzing the etiology of HNGN-PU in children and investigating the differences in clinical, laboratory, endoscopic, and histopathologic findings of peptic ulcers according to etiology including *H. pylori* infection*,* gastrotoxic drugs, systemic diseases, eosinophilic gastroenteritis (EoGE), and idiopathic peptic ulcers.

## Methods

### Study population and data collection

Of 1694 children and adolescents aged 18 or less who visited the Department of Pediatric Gastroenterology in Seoul National University Bundang Hospital with upper gastrointestinal symptoms and underwent esophagogastroduodenoscopy with biopsy between July 2003 and April 2017, 255 children diagnosed with peptic ulcers on endoscopy were recruited. The study subjects were categorized into 5 groups according to the etiology of the ulcer: 1) *H. pylori* infection (*n* = 51); 2) gastrotoxic drugs (*n* = 18); 3) idiopathic peptic ulcers (*n* = 144); 4) systemic diseases such as Henoch-Schönlein purpura (HSP) and Crohn’s disease (*n* = 23); and 5) EoGE (*n* = 19). Anastomosis site ulcer after gastrojejunostomy was excluded from the study. Patients referred from other hospitals after upper endoscopy and those transferred after ulcer treatment were also excluded.

Medical records of participants including demographic data, clinical features, allergy history, drug history, endoscopic findings, histopathologic findings, and laboratory tests were reviewed and analyzed retrospectively. Medication history was defined as drug intake during the 4-week period before diagnosis. Gastrotoxic drugs included NSAIDs or steroid usages, alkali ingestion, and disc battery ingestion.

This retrospective study was approved by the Institutional Review Board (IRB) of Seoul National University Bundang Hospital (IRB No. B-1707/409–105).

### Laboratory tests and radiologic investigations

All participants underwent laboratory tests including complete blood cell counts such as white blood cell count (WBC), absolute neutrophil count (ANC), eosinophil count, hemoglobin, hematocrit, platelet count, erythrocyte sedimentation rate (ESR), highly sensitive C-reactive protein (hsCRP), protein, albumin, liver function tests, serum amylase, lipase, iron panel, ferritin, urinalysis and urine cultures, stool examination for parasite, fecal occult blood, and fecal calprotectin levels, as well as abdominal X-ray and abdominal ultrasonography.

### Endoscopic evaluation

Esophagogastroduodenoscopy with mucosal biopsies was performed in all 255 study participants using a GIF-XP260 or GIF-Q260 scope (Olympus, Tokyo, Japan). Diagnosis of peptic ulcers was made based on endoscopic findings, which included the location, size, and number of ulcers and ulcer recurrence.

Colonoscopy with mucosal biopsies was performed additionally in 23 patients, to rule out gastric or duodenal ulcers due to systemic diseases.

### Histopathologic evaluation

Endoscopic mucosal biopsies were obtained from the esophagus, gastric antrum and body, and duodenum, during the upper gastrointestinal endoscopy, in all participants. Biopsy tissues were immediately fixed in formalin-filled bottle and processed to paraffin wax. Sections were cut at 3 μm and stained with hematoxylin-eosin and Wright-Giemsa stain for *H pylori*.

Modified Sydney classification was used to test for *H pylori* colonization, polymorphonuclear neutrophil activity, mononuclear cell infiltration, glandular atrophy, and intestinal metaplasia of the gastric tissue from the antrum and body. For the histopathologic diagnosis of *H pylori* infection, the specimens were subjected to rapid urease test in addition to histopathologic examination. *H pylori* infection was diagnosed if two endoscopic tests were positive for *H pylori*. For the rapid urease test kit (CLOtest, Ballard Inc., Draper, UT), the inoculated specimen was interpreted as positive if its color changed from yellow to pink within 24 h.

For the diagnosis of EoGE, tissue eosinophils were counted in 5 randomly selected high-power fields. Quantification of eosinophils was performed using an Axioskop40 microscope (Mirax-Carl Zeiss, Oberkochen, Germany) at 400x magnification. Histopathologic diagnosis of EoGE was made when the total number of infiltrating eosinophils per high power field was more than 15 in the esophagus and more than 20 in the stomach and duodenum without any other organic causes of tissue eosinophilia.

### Statistical analysis

Data for continuous parametric variables are presented as mean and standard deviations and that for categorical variables are presented as a percentage of the total number.

All statistical analyses were performed using PASW Statistics (SPSS version 22.0, SPSS Inc., Chicago, IL, USA). Continuous data were analyzed using the Mann-Whitney U test of two independent samples to compare two quantitative nonparametric variables and the Kruskal-Wallis test of independent samples to compare three or more quantitative nonparametric variables. *Chi*-square tests were used to compare categorical variables.

For statistical analysis of the differences in nonparametric continuous variables between the two groups, the Mann-Whitney test was additionally applied. The Bonferroni correction method was used to test and verity the variables marked as having differences. The significance level was divided by the number of comparison [α = 0.005 (0.05/10)] and then the Mann-Whitney test was performed.

For all statistical analyses, a two-sided *p* value < 0.05 was considered statistically significant.

## Results

### Patient characteristics of children with peptic ulcers

Totally 255 (15%) children out of 1694 patients who had undergone upper endoscopy for investigation of upper gastrointestinal symptoms, during the study period, were identified to have gastric or duodenal ulcers, on endoscopy, among whom one patient with anastomosis site ulcer was excluded from the study. These 255 children included 157 boys and 98 girls, with a mean age of 10.0 ± 5.1 years (range; 1 day ~ 18.0 years).

Of the 255 pediatric patients with peptic ulcers, 159 (62.4%) and 69 (27.1%) patients had only duodenal ulcers and gastric ulcers, respectively. Both gastric and duodenal ulcers were observed in 27 (10.6%) patients and multiple ulcers were observed in 73 (28.6%) patients, on endoscopy.

Regarding the etiology of peptic ulcers, *H. pylori* infection was diagnosed in 51 (20.0%) of the 255 patients; history of preceding gastrotoxic drugs intake in 18 (7.1%); systemic diseases such as Crohn’s disease and HSP in 23 (9.0%); EoGE-related peptic ulcers in 19 (7.5%); and idiopathic peptic ulcers in 144 (56.4%) patients (Table 1). EoGE ulcers accounted for 10.2% of 186 HNGN-PU in pediatric patients.

**Table 1:** Comparison of clinical features, endoscopic and histopathologic findings of children with peptic ulcers according to the etiology of ulcer

Variable	*H. pylori* infection (*n* = 51)	Gastrotoxic drug (*n* = 18)	Idiopathic peptic ulcer (*n* = 144)	Systemic disease (*n* = 23)	Eosinophilic gastroenteritis (*N* = 19)	*P* value*
**Clinical features**						
Male gender	34 (66.7)	7 (38.9)	86 (59.7)	15 (65.2)	15 (78.9)	
Age, years	14.3 (2.0–17.9)	6.0 (1.5–17.5)	9.1 (1 day-17.6)	8.6 (1.5–4.7)	13.6 (5.4–17.4)	**< 0.001**
Allergic history						
AD	0	0	4 (2.8)	1 (4.3)	5 (26.3)	**0.002**
AR	1 (4.3)	1 (7.7)	8 (9.4)	0	2 (14.3)	0.603
Asthma	1 (4.3)	1 (7.7)	1 (1.2)	0	0	0.305
Complications	6 (11.8)	1 (5.6)	10 (6.9)	1 (4.3)	3 (15.8)	0.742
Bleeding	4 (7.8)	1 (5.6)	7 (4.9)	1 (4.3)	2 (10.5)	0.940
Obstruction	2 (3.9)	0	1 (0.7)	0	1 (5.3)	0.424
Perforation	0	0	2 (1.4)	0	0	0.770
Ulcer recurrence	5 (9.8)	0	3 (2.1)	3 (13)	4 (21.1)	**0.016**
**Endoscopic findings n, (%)**						
Gastric ulcer only	10 (19.6)	11 (61.1)	42 (29.2)	4 (17.4)	2 (10.5)	**0.005**
Duodenal ulcer only	34 (66.7)	3 (16.7)	92 (63.9)	15 (65.2)	15 (78.9)	**< 0.001**
Both gastric and duodenal ulcers	7 (13.7)	4 (22.2)	10 (6.9)	4 (17.4)	2 (10.5)	0.427
Multiple ulcers	9 (17.6)	9 (50)	40 (27.8)	11 (47.8)	4 (21.1)	**0.023**
Gastric nodularity	27 (52.9)	0	8 (5.6)	1 (4.3)	5 (26.3)	**< 0.001**
Duodenal Nodularity	2 (3.9)	0	8 (5.6)	0	0	0.519
**Tissue eosinophils (count/HPF)** n, (range)						
Upper esophagus	0 (0–40)	0 (0)	0 (0–4)	0 (0–3)	0 (0–103)	**< 0.001**
Lower esophagus	0 (0–27)	0 (0)	0 (0–24)	0 (0)	0 (0–100)	**< 0.001**
Stomach antrum	0 (0–18)	0 (0–2)	0 (0–20)	9 (0–12)	5 (0–168)	**< 0.001**
Stomach body	0 (0–18)	0 (0–2)	0 (0–20)	0 (0–8)	1 (0–55)	**< 0.001**
Duodenal bulb	0 (0–53)	0 (0–10)	0 (0–18)	9 (0–6)	35 (0–84)	**< 0.001**
Duodenal 2nd	0 (0–42)	0 (0–10)	0 (0–19)	0 (0–25)	25 (0–100)	**< 0.001**

Data are expressed as number (%) for categorical variables or median (range) for continuous variables

*AD* atopic dermatitis, *AR* allergic rhinitis, *EoGE* eosinophilic gastroenteritis, *HPF* high power field

*P value less than 0.05 was set to be statistically significant

### Changes in the diagnosis of causes of peptic ulcers in children during the study period

**Fig. 1:**
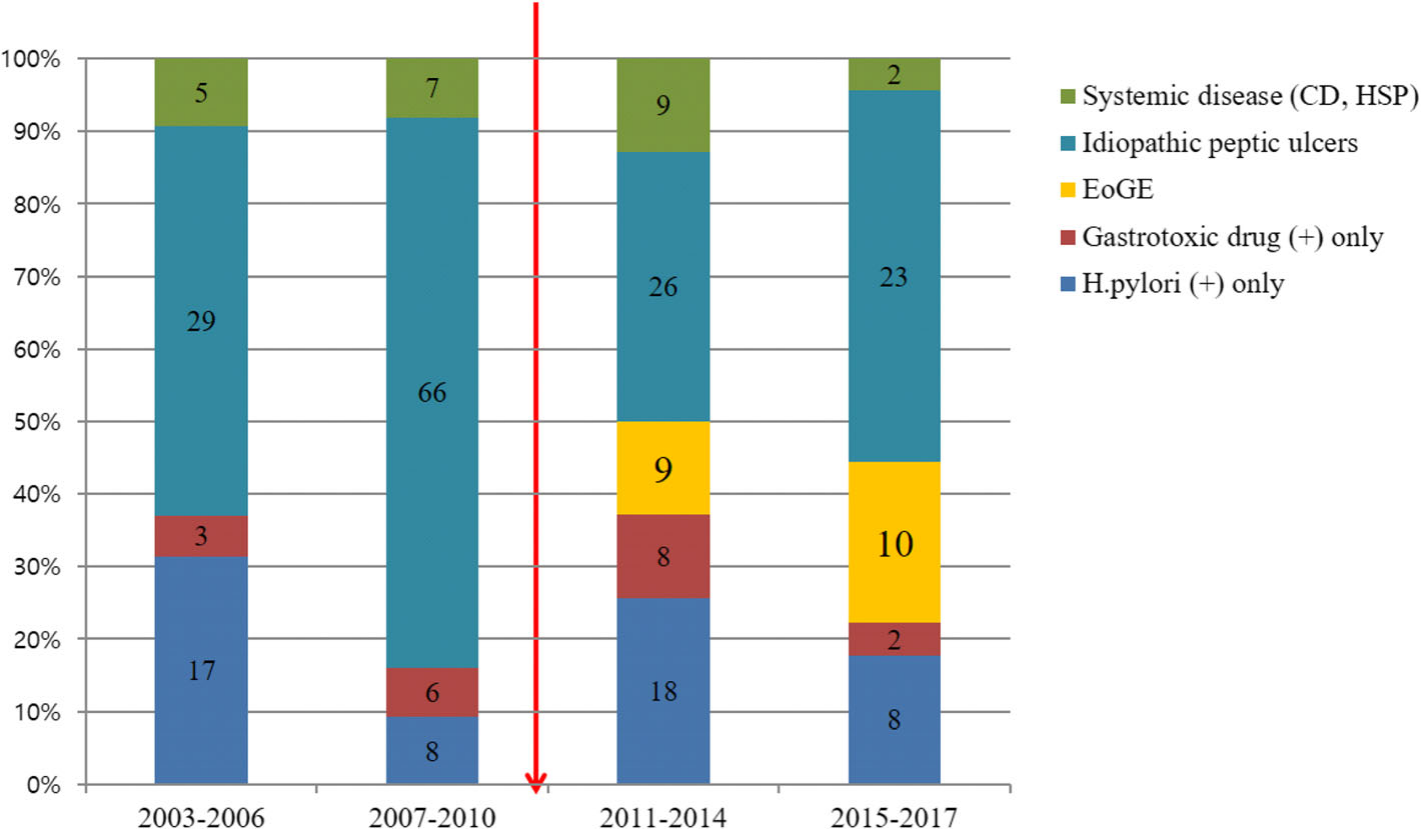
Distribution of the etiology of peptic ulcers in children by period. There is a change in the etiology of non-*H. pylori*and non-gastrotoxic drug ulcers since the new endoscopic biopsy protocol was implemented in 2011 to count tissue eosinophils in all specimens and eosinophilic gastroenteritis newly emerged as a cause of peptic ulcers in children.CD, Crohn’s disease; EoGE, eosinophilic gastroenteritis; HSP, Henoch-Schönlein purpura

Figure 1 reveals the distribution of the etiology of peptic ulcers in children during the study period from 2003 to 2017. Since the introduction of a new protocol for histopathologic evaluation of endoscopic biopsy to count tissue eosinophils in all specimens in 2011, the distribution of causes of HNGN-PU changed and EoGE newly emerged as a cause of peptic ulcers in children (Fig. 1).

### Comparison of clinical and laboratory factors among the 5 groups according to the etiology of peptic ulcers in children

Regarding clinical factors related to peptic ulcers in children, age at diagnosis (*p* < 0.001), history of atopic dermatitis (*p* = 0.002), and ulcer recurrence (*p* = 0.016) were significantly different among the 5 peptic ulcer groups (Table 1). When applying the post-test analysis between the two groups, statistically significant differences were found between the *H. pylori* infection group and the idiopathic peptic ulcer group at the significance level of 0.005 (Supplement 1). Idiopathic peptic ulcer group showed statistically significant differences in atopic dermatitis history, duodenal ulcer, and peripheral eosinophilia compared to *H. pylori* infection group.

Regarding laboratory factors, WBC counts (*p* = 0.008), eosinophil counts in blood (*p* = 0.013), platelet counts (*p* < 0.001), serum levels of albumin (*p* = 0.027), iron (*p* < 0.001), ESR (*p* < 0.001), and hsCRP (*p* = 0.016) differed significantly among the 5 ulcer groups (Table 2).

**Table 2:** Comparison of laboratory findings of children with peptic ulcers according to the etiology of ulcer

Variable, n (%)	*H. pylori* infection (*N* = 51)	Gastrotoxic drug (*N* = 18)	Idiopathic peptic ulcer (*N* = 144)	Systemic disease (*N* = 23)	Eosinophilic gastroenteritis (*N* = 19)	*P* value*
WBC (×103/μl)	6.5 (3.2–13.3)	7.7 (4.5–26.1)	7.4 (3.3–29.7)	8.9 (5–18.9)	6.4 (4.2–10.3)	**0.008**
ANC (μl)	3644 (1450-10,600)	3161 (1663 – 22,933)	3649 (521–18,680)	4706 (1268 –16,795)	3157 (1819 –6874)	0.064
Eosinophil (%)	2 (0.6–12.1)	2.25 (0.1–5.7)	2.4 (0–13.4)	1.4 (0.1–6.3)	4.5 (1.3–22.5)	**0.009**
Hematocrit (%)	38.8 (18.8–49.9)	39.1 (31.2–43.3)	39.5 (22.3–58.3)	27.6 (26.0–47.5)	40 (22.3–45.9)	0.395
Hemoglobin (g/dL)	12.9 (8.0–16.4)	12.9 (9.9–14.3)	13.3 (6.2–20.3)	12.5 (8.8–15.6)	13.5 (7.5–15.3)	0.303
Platelet (× 103/μl)	276 (133–579)	348 (115–470)	278 (10–574)	382 (150–560)	296 (135–577)	**0.000**
Protein (g/dL)	7.1 (5.0–8.3)	6.9 (6.1–7.7)	7.0 (4.2–8.5)	6.8 (5.0–9.0)	6.9 (6.3–7.7)	0.734
Albumin (g/dL)	4.5 (3.0–5.0)	4.2 (3.3–5.0)	4.4 (2.4–5.4)	3.8 (3.2–4.8)	4.4 (3.8–5.5)	**0.027**
Iron (μg/dL)	57 (10–172)	85 (26–107)	78.5 (9–208)	27.5 (12.0–126.0)	64.5 (12.0–199)	**0.001**
Ferritin (ng/ml)	28.8 (1.9–99.0)	49.2 (32.4–81.0)	43.7 (6–217)	17.9 (4.0–183.8)	44.0 (4–164)	0.055
TIBC (μg/dL)	388 (280–580)	329.5 (220–459)	358 (188–528)	363.5 (248.0–486.0)	265 (251–463)	0.506
ESR (mm/hr)	6 (2–52)	11.0 (2–17)	4 (2–79)	25.5 (4.0–59.0)	4 (2–21)	**0.000**
hsCRP (mg/dL)	0.02 (0–18.4)	0.3 (0–2.6)	0.12 (2–24.6)	1.3 (0–4.4)	0.02 (0–5.3)	**0.016**

Data are expressed as median (range)

*ANC* absolute neutrophil count, *ESR* erythrocyte segmentation rate, *hsCRP* highly sensitive C-reactive protein, *TIBC* total iron binding capacity, *WBC* white blood cell

*P value less than 0.05 was set to be statistically significant

### Comparison of endoscopic and histopathologic findings among the 5 groups according to the etiology of peptic ulcers in children

Regarding endoscopic findings, gastric ulcers (*p* = 0.005), duodenal ulcers (*p* < 0.001), multiple ulcers (*p* = 0.023), and gastric mucosal nodularity (*p* < 0.001) differed significantly among the 5 peptic ulcer groups (Table 1). Gastric ulcers were most frequently observed on upper endoscopy in the gastrotoxic drug group (61.1%), followed by the idiopathic ulcer group (29.2%) (Table 1). Duodenal ulcers were most frequently noted in the EoGE group (78.9%), followed by the *H. pylori* group (66.7%) and the systemic disease group (65.2%) (Table 1). According to the results of the post-test for duodenal ulcer, there was a statistically significant difference between the *H. pylori* infection group and the idiopathic peptic ulcer group at the significance level of 0.005 (Supplement 1). Gastric mucosa nodularity was most frequently noted in the *H. pylori* group (52.9%), followed by the EoGE group (26.3%) (Table 1).

Regarding histopathologic findings of upper gastrointestinal tracts, tissue eosinophil counts were significantly different in the esophagus, stomach, and duodenum among the 5 ulcer groups (all *p* < 0.001) (Table 1).

### Comparison of EoGE ulcers with non-EoGE ulcers

When clinical features of EoGE ulcers were compared with those of the other groups, the patients with EoGE ulcers were significantly older in age (median 13.6 yrs. vs. 10.2 years, *p* = 0.022) and revealed higher rates of ulcer recurrence (21.1% in EoGE group vs. 4.7% in non-EoGE group, *p* = 0.018), and atopic dermatitis history (26.3% in EoGE group vs. 2.1% in non-EoGE group, *p* = 0.001) (Tables 3 and 4).

**Table 3:** Comparison of clinical features, endoscopic findings and tissues eosinophilia between the eosinophilic gastroenteritis (EoGE) ulcer and the non-EoGE ulcer group

Variable	Non-EoGE (*N* = 236)	EoGE (*N* = 19)	*P* value*
**Clinical features**			
Male gender (%)	142 (60.2)	15 (78.9)	0.105
Age, years	10.2 (1–18 days)	13.6 (5.4–17.4)	**0.022**
Complications	18 (7.6)	3 (15.8)	0.197
Bleeding	13 (5.5)	2 (10.5)	0.309
Obstruction	3 (1.3)	1 (5.3)	0.268
Perforation	2 (0.8)	0	1.000
Ulcer recurrence	11 (4.7)	4 (21.1)	**0.018**
Allergic history			
Atopic dermatitis	5 (2.1)	5 (26.3)	**< 0.001**
Allergic rhinitis	10 (7.2)	2 (14.3)	0.305
Asthma	3 (2.2)	0	1.000
**Endoscopic findings** n, (%)			
Gastric ulcer only	67 (28.4)	2 (10.5)	0.092
Duodenal ulcer only	144 (61.0)	15 (78.9)	0.121
Gastric and Duodenal ulcer	25 (10.5)	2 (10.5)	1.000
Multiple ulcers	69 (29.2)	4 (21.1)	0.448
Stomach nodularity	36 (15.3)	5 (26.3)	0.202
Duodenal nodularity	10 (4.2)	0	1.000
**Tissue eosinophils (count/HPF)** n, (range)			
Upper esophagus	0 (0–4)	0 (0–103)	**< 0.001**
Lower esophagus	0 (0–24)	0 (0–100)	**< 0.001**
Stomach, antrum	0 (0–20)	5 (0–168)	**< 0.001**
Stomach, body	0 (0–20)	1 (0–55)	**< 0.001**
Duodenal bulb	0 (0–18)	35 (0–84)	**< 0.001**
Duodenal 2nd portion	0 (0–42)	25 (0–100)	**< 0.001**

Data are expressed as Data are expressed as number (%) for categorical variables or median (range) for continuous variables

*EoGE* eosinophilic gastroenteritis, *HPF* high power field

*P value less than 0.05 was set to be statistically significant

**Table 4:** Comparison of laboratory findings between the eosinophilic gastroenteritis (EoGE) ulcer and the non-EoGE ulcer group

Variable	Non-EoGE (*N* = 236)	EoGE (*N* = 19)	*P* value
WBC (×103/μl)	7.47 (3.2–29.7)	6.4 (4.2–10.3)	0.130
ANC (μl)	3710 (521–22,933)	3157 (1819-6874)	0.106
Eosinophil (%)	2.2 (0–13.4)	4.5 (1.3–22.5)	**0.001**
Hematocrit (%)	38.9 (18.8–58.3)	40 (22.3–45.9)	0.734
Hemoglobin (g/dL)	13.2 (6.2–20.3)	13.5 (7.5–15.3)	0.707
Platelet (×103/μl)	283 (10–579)	296 (135–577)	0.532
Protein (g/dL)	7.0 (4.2–9.0)	6.9 (6.3–7.7)	0.670
Albumin (g/dL)	4.4 (2.4–5.4)	4.4 (3.8–5.5)	0.683
Iron (μg/dL)	71 (9.0–208)	64.5 (12.0–199)	0.903
Ferritin (ng/ml)	37 (1.9–217)	44.0 (4–164)	0.848
TIBC (μg/dL)	365.5 (188–580)	265 (251–463)	0.997
ESR (mm/hr)	6 (2–79)	4 (2–21)	0.120
hsCRP (mg/dL)	0.14 (0–24.6)	0.02 (0–5.3)	0.059

Data are expressed as median (range)

*ANC* absolute neutrophil count, *EoGE* eosinophilic gastroenteritis, *ESR* erythrocyte segmentation rate, *hsCRP* highly sensitive C-reactive protein, *TIBC* total iron binding capacity, *WBC* white blood cell

*P value less than 0.05 was set to be statistically significant

When comparing the laboratory, endoscopic, and histopathologic findings of the EoGE group and the non-EoGE ulcer groups, only blood eosinophil counts and tissue eosinophil counts of the esophagus, stomach, and duodenum were significantly higher in the EoGE group (*p* = 0.001 & *p* < 0.001, respectively) (Supplemental Digital Content 1 & Table 3).

## Discussion

This is the first study that investigated the etiology of peptic ulcers in pediatric patients and its change over time, especially emphasizing on HNGN-PU as an emerging etiology of peptic ulcers and EoGE as a significant cause of HNGN-PU in children.

Previously, *H. pylori* infection and gastrotoxic drugs were considered the main causes of peptic ulcers [1–4]. In developing countries including South Korea, the prevalence of *H. pylori* infection has increased to 50% of the general population, and in 15% of the infected patients, *H. pylori* infection led to the development of peptic ulcers. Additionally, *H. pylori-*negative duodenal ulcers were reported in several studies in the early 1990s in developed countries [12, 20, 21], accounting for 2.8–6.0% of peptic ulcers and half of NSAID-associated ulcers [20, 21]. Previous studies on the etiology of peptic ulcers have reported that idiopathic duodenal ulcers not associated with *H. pylori* or NSAIDs (HNGN-PU) were rare in the past.

However, in the recent years, the proportion of HNGN-PU among causes of peptic ulcers has been increasing gradually, due to decrease in the prevalence of *H. pylori* infection, because of effective prevention by environment improvement and antibiotic treatments, and increase in the proper use of NSAIDs [9–11, 22, 23]. Recent studies have showed that the prevalence of idiopathic peptic ulcers (e.g. HNGN-PU) was higher than that which was reported by previous studies [6, 16]. Moreover, high prevalence of HNGN-PU in adults was recently reported in the USA and Australia [6]. In addition, there have been several case reports that peptic ulcers did not improve or recurred after *H. pylori* eradication. Furthermore, according to Laine et al., about 20% of duodenal ulcers recurred 6 months after successful eradication of *H. pylori,* in a meta-analysis of seven well-designed trials [13]. Charpignon et al. demonstrated that there were significant differences in the age of onset and the comorbidity of idiopathic peptic ulcer diseases (e.g. HNGN-PU), compared with both *H. pylori* and NSAIDs/aspirin-associated peptic ulcers [14]. However, the prevalence and clinical features of HNGN-PU in children has not been evaluated yet.

In the present study conducted for 14 years from July 2003 to April 2017, we recruited children with peptic ulcers and categorized them into 5 groups to investigate the differences in clinical features and the laboratory, endoscopic, and histopathologic findings of peptic ulcers in children, according to the etiology. Our study revealed that the proportion of *H. pylori* infection-associated ulcers and NSAIDs/aspirin-associated peptic ulcers were 20 and 7.1%, respectively, in pediatric patients. The prevalence of *H. pylori* infection in Korean children declined to less than 10% in 2000, implying that *H. pylori* is no longer the main cause of peptic ulcers. The notable point of our study results is that the proportion of HNGN-PU including idiopathic peptic ulcers, systemic diseases (Crohn’s disease, HSP), and EoGE was relatively high, with 186 of 255 peptic ulcer cases (72.9%) in children.

To date, most causes of HNGN-PU in children are unknown. EoGE is an etiology of HNGN-PU in adult patients and there have been studies stating that EoGE may be a cause of refractory peptic ulcer and several case reports of EoGE presenting with refractory peptic ulcer or perforated duodenal ulcer in both adults and children [24–26]. However, there were no studies on EoGE as a possible cause of HNGN-PU in children yet. In the present study, HNGN-PU was found to be relatively prevalent in children with peptic ulcer throughout the study period, and then EoGE emerged as a cause of HNGN-PU since we introduced a new endoscopic biopsy protocol to count tissue eosinophils in all specimens in 2011. In our study, 19 of 255 (7.5%) patients with peptic ulcers were recently diagnosed with EoGE on the basis of clinical features and histopathologic findings of significant tissue eosinophil infiltrations, without any other organic diseases associated with gastrointestinal eosinophilia. No cases were diagnosed as EoGE ulcers before the year of 2011. Thus, the overall proportion of EoGE ulcers accounted for 10.2% of 186 HNGN-PU in pediatric patients, which was 0% before 2011. All of these 19 EoGE cases were detected using the protocol of full tissue eosinophil evaluation by pathologists, without additional hospital costs.

In this study, increased blood eosinophil counts suggested EoGE, as if an increase in platelet, ESR, and hsCRP counts implied systemic diseases. This is consistent with previous reports that 50% of patients with EoGE disease had peripheral eosinophilia [17]; EoGE ulcer is a significant laboratory finding that cause suspicions. And EoGE among the 5 ulcer groups had more duodenal ulcers than gastric ulcers in our study. In the gastrotoxic drug group, however, multiple gastric ulcers were found to be more common. Notably, 52.9% of *H pylori* infection and 26.3% of EoGE were expressed as nodular gastritis. Therefore, EoGE as well as *H pylori* infection should be included in the differential diagnosis of nodular gastritis and duodenal ulcers in endoscopic findings.

This study has a few limitations. In a retrospective study, eosinophils cannot be counted through biopsy from the beginning of the study. And since there is no accurate drug history in some patients, it is likely that the gastrotoxic drug group has been misclassified as idiopathic group. In addition, the limitation of accurate diagnosis of *H. pylori* infection can also be a problematic issue that may increase the prevalence of HNGN-PU [27]. Since the diagnostic criteria for EoGE are still controversial, there is a possibility of overdiagnosis. A prospective study focusing on EoGE and consensus on diagnostic criteria is required in the future.

## Conclusions

Our study revealed that the prevalence of EoGE ulcers in pediatric patients with HNGN-PU was 10.2%, much higher than previously reported. In children with HNGN-PU, ulcer recurrence, peripheral eosinophilia, and history of atopic dermatitis might provide high levels of clinical suspicion for EoGE, requiring thorough histopathologic investigation of tissue eosinophils counts, on the basis of endoscopic biopsy.

## Supplementary information

**Additional file 1: Supplement 1.** Post-test, multiple comparison analysis of clinical features, endoscopic, histopathological, and laboratory findings in children with peptic ulcer according to the etiology of ulcer.

## Data Availability

The datasets used and/or analyzed during the current study are available from the corresponding author on reasonable request.

## Abbreviations

*H. pylori*: *Helicobacter pylori*
HNGN-PU: *H. pylori*-negative and/or gastrotoxic drug-negative peptic ulcers (HNGN-PU)
EoGE: Eosinophilic gastroenteritis
NSAIDs: Non-steroidal anti-inflammatory drugs
HSP: Henoch-Schönlein purpura
WBC: White blood cell count
ANC: Absolute neutrophil count
ESR: Erythrocyte sedimentation rate
hsCRP: Highly sensitive C-reactive protein
