# Supplementary material for: Eosinophilic gastroenteritis as a cause of non-Helicobacter pylori, non-gastrotoxic drug ulcers in children
Source: BMC Gastroenterol. 2020 Aug 20;20:280. doi: 10.1186/s12876-020-01416-7 (PMC7439514; doi:10.1186/s12876-020-01416-7)
Supplement: Supplementary file 1 — Additional file 1: Supplement 1. Post-test, multiple comparison analysis of clinical features, endoscopic, histopathological, and laboratory findings in children with peptic ulcer according to the etiology of ulcer. [file 12876_2020_1416_MOESM1_ESM.docx]

**Supplement 1.** Post-test, multiple comparison analysis of clinical features, endoscopic, histopathological, and laboratory findings in children with peptic ulcer according to the etiology of ulcer

|  | *H. pylori* infection  (N=51) | Gastrotoxic drug  (N=18) | Idiopathic peptic ulcer  (N=144) | Systemic disease  (N=23) | Eosinophilic gastroenteritis  (N=19) | A vs B | A vs C | A vs D | A vs E | B vs C | B vs D | B vs E | C vs D | C vs E | D vs E |
| --- | --- | --- | --- | --- | --- | --- | --- | --- | --- | --- | --- | --- | --- | --- | --- |
| ***Clinical features*** | | | | | | | | | | | | | | | |
| age | 14.3  (2.0-17.9) | 6.0  (1.5-17.5) | 9.1  (1 -17.6) | 8.6  (1.5-4.7) | 13.6  (5.4-17.4) | * | * | * | ns | ns | ns | * | ns | * | * |
| AD | 0 | 0 | 4 (2.8) | 1 (4.3) | 5 (26.3) | ns | ns | ns | * | ns | ns | ns | ns | * | ns |
| Recurrence | 5 (9.8) | 0 | 3 (2.1) | 3 (13) | 4 (21.1) | ns | * | ns | ns | ns | ns | ns | ns | * | ns |
| ***Endoscopic findings*** | | | | | | | | | | | | | | | |
| Gastric ulcer only | 10 (19.6) | 11 (61.1) | 42 (29.2) | 4 (17.4) | 2 (10.5) | * | ns | ns | ns | ns | * | * | ns | ns | ns |
| Duodenal  ulcer only | 34 (66.7) | 3 (16.7) | 92 (63.9) | 15 (65.2) | 15 (78.9) | * | ns | ns | ns | * | * | * | ns | ns | ns |
| Multiple  ulcers | 9 (17.6) | 9 (50) | 40 (27.8) | 11 (47.8) | 4 (21.1) | ns | ns | ns | ns | ns | ns | ns | ns | ns | ns |
| Gastric  nodularity | 27 (52.9) | 0 | 8 (5.6) | 1 (4.3) | 5 (26.3) | * | * | * | ns | ns | ns | ns | ns | * | ns |
| **Tissue eosinophils** (count/*HPF*) | | | | | | | | | | | | | | | |
| Upper esophagus | 0 (0-40) | 0 (0) | 0 (0-4) | 0 (0 – 3) | 0 (0-103) | ns | ns | ns | * | ns | ns | ns | ns | * | ns |
| Lower esophagus | 0 (0-27) | 0 (0) | 0 (0-24) | 0 (0) | 0 (0-100) | ns | ns | ns | * | ns | ns | ns | ns | * | ns |
| Stomach  antrum | 0 (0-18) | 0 (0-2) | 0 (0-20) | 9 (0 – 12) | 5 (0-168) | ns | ns | ns | * | ns | ns | * | ns | * | * |
| Stomach body | 0 (0-18) | 0 (0-2) | 0 (0-20) | 0 (0 – 8) | 1 (0-55) | ns | ns | ns | ns | ns | ns | ns | ns | * | ns |
| Duodenal  bulb | 0 (0-53) | 0 (0-10) | 0 (0-18) | 9 ( 0 – 6) | 35 (0-84) | ns | ns | ns | * | ns | ns | ns | ns | * | * |
| Duodenal 2nd | 0 (0-42) | 0 (0-10) | 0 (0-19) | 0 (0 – 25) | 25 (0-100) | ns | ns | ns | * | ns | ns | ns | ns | * | * |
| ***Laboratory findings*** | | | | | | | | | | | | | | | |
| WBC (x10^3^/㎕) | 6.5  (3.2-13.3) | 7.7  (4.5-26.1) | 7.4  (3.3-29.7) | 8.9  (5–18.9) | 6.4  (4.2-10.3) | ns | ns | * | ns | ns | ns | ns | ns | ns | * |
| Eosinophil (%) | 2  (0.6-12.1) | 2.25  (0.1-5.7) | 2.4  (0-13.4) | 1.4  (0.1–6.3) | 4.5  (1.3-22.5) | ns | ns | ns | * | ns | ns | ns | ns | * | * |
| Platelet (x10^3^/㎕) | 276  (133- 579) | 348  (115 - 470) | 278  (10-574) | 382  (150–560) | 296  (135-577) | ns | ns | * | ns | ns | ns | ns | * | ns | ns |
| Albumin (g/dL) | 4.5  (3.0-5.0) | 4.2  (3.3 - 5.0) | 4.4  (2.4-5.4) | 3.8  (3.2–4.8) | 4.4  (3.8-5.5) | ns | ns | * | ns | ns | ns | ns | * | ns | * |
| Iron (μg/dL) | 57  (10-172) | 85  (26 - 107) | 78.5  (9-208) | 27.5  ( 12.0–126.0) | 64.5  (12.0 -199) | ns | ns | ns | ns | ns | ns | ns | * | ns | ns |
| ESR (mm/hr) | 6 (2-52) | 11.0 (2-17) | 4 (2-79) | 25.5 (4.0–59.0) | 4 (2-21) | ns | ns | * | ns | ns | * | ns | * | ns | * |
| hsCRP (mg/dL) | 0.02 (0-18.4) | 0.3 (0 - 2.6) | 0.12 (2-24.6) | 1.3 (0–4.4) | 0.02 (0-5.3) | ns | ns | ns | ns | ns | ns | ns | ns | ns | * |

AD, atopic dermatitis; AR, allergic rhinitis; ANC, absolute neutrophil count; ESR, erythrocyte segmentation rate; HPF, high power field; hsCRP, highly sensitive C-reactive protein; TIBC, total iron binding capacity; WBC, white blood cell; vs, versus

A, *H. pylori* infection group; B, Gastrotoxic drug group; C, Idiopathic peptic ulcer group; D, Systemic disease group; E, Eosinophilic gastroenteritis group

Data are expressed as number (%) for categorical variables or median (range) for continuous variables.

**p* < 0.005 as Bonferroni-corrected significance level for multiple comparison

ns, not significant, *p* > 0.005
